# Mathematical modeling of dormant cell formation in growing biofilm

**DOI:** 10.3389/fmicb.2015.00534

**Published:** 2015-05-28

**Authors:** Kotaro Chihara, Shinya Matsumoto, Yuki Kagawa, Satoshi Tsuneda

**Affiliations:** ^1^Department of Life Science and Medical Bioscience, Waseda UniversityTokyo, Japan; ^2^Center for Biofilm Engineering, Montana State UniversityBozeman, MT, USA; ^3^Institute for Nanoscience and Nanotechnology, Waseda UniversityTokyo, Japan

**Keywords:** biofilm, dormancy, persistence, individual-based modeling, mathematical model

## Abstract

Understanding the dynamics of dormant cells in microbial biofilms, in which the bacteria are embedded in extracellular matrix, is important for developing successful antibiotic therapies against pathogenic bacteria. Although some of the molecular mechanisms leading to bacterial persistence have been speculated in planktonic bacterial cell, how dormant cells emerge in the biofilms of pathogenic bacteria such as *Pseudomonas aeruginosa* remains unclear. The present study proposes four hypotheses of dormant cell formation; stochastic process, nutrient-dependent, oxygen-dependent, and time-dependent processes. These hypotheses were implemented into a three-dimensional individual-based model of biofilm formation. Numerical simulations of the different mechanisms yielded qualitatively different spatiotemporal distributions of dormant cells in the growing biofilm. Based on these simulation results, we discuss what kinds of experimental studies are effective for discriminating dormant cell formation mechanisms in biofilms.

## Introduction

In natural environments or within the tissues of living organisms, some bacterial species form biofilms to ensure their long-term survival (Costerton et al., 1999; Hall-Stoodley et al., 2004). Bacteria attached to solid surfaces form mature biofilms by proliferating and producing extracellular polymeric substance (EPS), which captures planktonic bacteria (Stewart and Franklin, 2008). The EPS is composed of DNA molecules, proteins and polysaccharide. The thick biofilm structure impedes antibiotic diffusion and reduces the mobility of immune cells. Therefore, biofilms are responsible for the chronic and intractable characteristics of bacterial infectious disease (Stewart and Costerton, 2001; Stewart, 2002), which increase the morbidity and mortality of infections in immunocompromised patients. For example, intractable *Pseudomonas aeruginosa* infections have been reported in cystic fibrosis patients (Harrison, 2007).

One aspect of chronic characteristics of bacterial infectious disease is persister cells, which can tolerate sublethal concentrations of antibiotics. Unlike antibiotic resistant cells that carry genetic mutations, persister cells are nonheritable phenotypic variants. Some persister cells suppress their metabolism, including cell membrane formation, protein synthesis, and DNA replications (Lewis, 2007; Lewis et al., 2010). Such dormant bacteria can survive antibiotic exposure because their antibiotic target sites are deactivated. Actually, Balaban et al. (2004) investigated the single cell dynamics of the high persistence (*hip*) mutants (Moyed and Bertrand, 1983) of *Escherichia coli* by using microfluidic devices and found that preexisting subpopulations having reduced growth rates showed persistence under ampicillin exposure.

Several mechanisms of dormant cell formation have been proposed (Maisonneuve and Gerdes, 2014). Elowitz et al. (2002) posited that bacterial gene expressions related to physiological states are stochastically activated or inactivated. Expansion of this stochastic gene noise induces the formation of the stable subpopulation of some different bacterial phenotypes, such as dormant cell and active cell (Balaban et al., 2004). However, whether dormant cell is formed stochastically in a biofilm is unclear because previous studies were conducted by use of planktonic bacterial cell. Other researchers indicated that low nutrient concentration and diauxic shift of carbon initiate the ppGpp-controlled stress response, which activates the signal pathways for cell dormancy in planktonic bacterial cell (Nguyen et al., 2011; Amato et al., 2013). In a more recent study, Wakamoto et al. (2013) investigated persister cell dynamics of *Mycobacterium smegmatis* at a single cell level by microfluidic device in the presence of the drug isoniazid (INH) and time-lapse microscopy measurement. They showed that all persister cells did not necessarily repress their division, and persister cell did not always include dormant cell in the INH disposal of *M. smegmatis*, whereas dormant cell always included persister cell. Moreover, they showed that persister cells appeared due to the genetic stochastic expression encoded catalase-peroxidase (KatG), which activates INH.

During the biofilm formation, bacteria consume nutrients and oxygen, creating oxygen and nutrient concentration gradients within the developing biofilm (Stewart and Franklin, 2008). Therefore, dormant cells will emerge from the bottom of the biofilms, where the nutrient and oxygen are depleted to induce the dormant state due to the direct effect of nutrient or oxygen limitation, or the indirect effect of time-dependent growth arrest. To prevent the emergence of dormant cells, we need to elucidate the dynamics of dormant cell formation in growing biofilms. However, the spatiotemporal dynamics by which dormant cells emerge in growing biofilms are difficult to investigate, because experimental methods for specifically detecting dormant cells are not sufficiently developed. Consequently, which mechanisms of dormancy; stochastic, nutrient limitation, oxygen limitation, and time-dependent growth arrest, has a major impact against the bacterial dormancy within growing biofilm is unknown.

Experiments can be complemented by mathematical modeling, which has become one of the most promising tools for studying the emergence of dormant cells in growing biofilms. Many mathematical models of biofilm formation in specific environments have been developed. Multi-species biofilm formation has been modeled by one-dimensional partial differential equations describing a reaction–diffusion system (Rittmann et al., 2002). Other researchers have adopted two- and three-dimensional cellular automaton algorithms that replicate the complex structures of porous biofilms, such as mushroom-like structures with many voids and channels (Picioreanu et al., 1998a,b). An individual-based modeling (IbM) approach originally proposed for bacterial colony growth (Kreft et al., 1998), in which bacterial cells were represented as hard spheres, has been adapted to biofilms and microbial granule modeling by allowing continuous displacements and directions of the biomass particles (Picioreanu et al., 2004; Matsumoto et al., 2010; Kagawa et al., 2015). The IbM algorithm is considered as a suitable basis for describing the dynamics of rare species such as dormant cells in growing biofilms.

Therefore, in the present study, we develop three-dimensional biofilm models based on the IbM algorithm to understand and predict the formation of dormant cells within growing biofilms. We propose four hypothetical mechanisms of dormant cell formation. Using the proposed model, we then simulate the spatiotemporal dynamics of dormant cell emergence under each hypothesis. Finally, we discuss what kinds of potential experimental design are effective for verifying the given hypotheses.

## Methods

We first constructed a three-dimensional biofilm model based on the IbM algorithm. Next, we implemented four hypothetical mechanisms of dormant cell formation in the model. Here we proposed that dormant cells were formed by stochastic, nutrient-dependent, oxygen-dependent, or time-dependent processes. Using the developed model, we numerically simulated the spatiotemporal formation of dormant cells in a growing biofilm. Particularly, we perturbed the bulk nutrient or oxygen concentration, and observed the changes in the dormant cell distribution throughout the biofilm. Finally, based on these simulation results, we discussed what kinds of experimental approaches are effective for discriminating the above-mentioned hypotheses. The discussed experiments should provide new insights into the mechanisms of dormant cell formation.

### Construction of a three-dimensional biofilm model

Our three-dimensional (3D) mathematical model of biofilm formation is based on the IbM algorithm (Picioreanu et al., 2007). Parameter values used in this model are summarized in Table 1. Each bacterial cell is represented as a sphere of radius *R* positioned at (*x, y, z*) in 3D space. The radius is given by:

(1) $$R={\left(3m/4\pi c\right)}^{1/3},$$

where *m* and *c* are the mass and density of the cells, respectively. Each particle undergoes the following three behaviors of real bacteria (Supplementary Figure 1A):

(i) *Growth*: Each bacterial cell consumes nutrient at a rate given by:

(2) $$q=q^{\text{max}}\cdot [S_{S}/(S_{S}+K_{S})]\cdot [S_{O}/(S_{O}+K_{O})],$$

where *q*^max^ is the maximum consumption rate, *S_S_* and *S_O_* are the local concentrations of the nutrient and oxygen, respectively, and *K_S_* and *K_O_* are the half-saturation constants of the nutrient and oxygen, respectively. Cells consume oxygen along with nutrient, and increase their masses according to the stoichiometric ratios defined in Tables 2, 3.

**Table 1:** **Parameter values used in 3D biofilm model**.

**Parameter**	**Symbol**	**Value**	**Unit**	**References**
Time step	Δ *t*	0.2	h	
System size	*L_x_, L_y_, L_z_*	2.0 × 10^−4^	m	Picioreanu et al., 2007
Grid number	*N, M, L*	33		
Thickness of boundary layer	*N*_bl_	3	Δ*x* (= *L_x_/N*)	This study
Maximum consumption rate of active cell	*q*^max^_A_	75	(gCOD/gCOD)/day	Picioreanu et al., 2007
Maximum consumption rate of dormant cell	*q*^max^_D_	15	(gCOD/gCOD)/day	This study
Maximum cell density	*C*	1.5 × 10^5^	gCOD/m^3^	Picioreanu et al., 2007
Maximum cell mass	*m*_max_	9.60 × 10^−12^	gCOD	This study
Initial cell mass	*m*_min_	4.80 × 10^−12^	gCOD	This study
Bulk concentration of oxygen	*C_O_*^bulk^	4.0	g/m^3^	Xavier et al., 2005
Bulk concentration of nutrient	*C_S_*^bulk^	100	gCOD/m^3^	Xavier et al., 2005
Diffusion coefficient of oxygen	*D_O_*	2.0 × 10^−4^	m^2^/day	Xavier et al., 2005
Diffusion coefficient of nutrient	*D_S_*	4.5 × 10^−6^	m^2^/day	Picioreanu et al., 2007
Half saturation constant of oxygen	*K_O_*	0.35	g/m^3^	Xavier et al., 2005
Half saturation constant of nutrient	*K_S_*	20	gCOD/m^3^	Picioreanu et al., 2007

**Table 2:** **Stoichiometric matrix and kinetic rate expressions**.

**Process**	**Solute species**		**Particle species**		**Rate**
	***S_S_***	***S_O_***	***X*_A_**	***X*_D_**	
Growth of active cell	−1	−(1-*Y*_A_)	*Y*_A_		*q*^max^_A_·[*S*_S_/(*S*_S_+*K*_S_)]·[*S*_O_/(*S*_O_+*K*_O_)]·X_A_
Growth of dormant cell	−1	− (1-*Y*_D_)		*Y*_D_	*q*^max^_D_·[*S*_S_/(*S*_S_+*K*_S_)]·[*S*_O_/(*S*_O_+*K*_O_)]·X_D_

**Table 3:** **Stoichiometric parameters for microbial reactions**.

**Parameters**	**Symbol**	**Value**	**Unit**	**Reference**
Yield on nutrient at active cell	*Y*_A_	0.5	gCOD/gCOD	Xavier et al., 2005
Yield on nutrient at dormant cell	*Y*_D_	0	gCOD/gCOD	This study

(ii) *Division*: When a cell reaches its maximum mass, it divides into two uniform daughter cells. The daughter cells do not overlap but remain attached, as described previously (Picioreanu et al., 2004).

(iii) *Shoving*: The growth and division processes cause overlapping of the spherical particles. To minimize these overlaps, each particle pushes its neighboring particles multiple times in the shoving algorithm (Picioreanu et al., 2004). Kagawa et al. (2014) related the number of shoves to the area of the overlap region in two-dimensional (2D) space. In the present study, the number of shoves was fixed at 200. Previous models considered bacterial decay, death and detachment processes (Xavier et al., 2005); these factors were excluded in the present model.

Movement of the particles obeys the following two boundary conditions: (a) particles cannot penetrate into the bottom surface of the system, and (b) periodic boundary conditions are imposed at the lateral boundaries.

The spatial distributions of the nutrient and oxygen concentrations were calculated by the following reaction–diffusion equations:

(3) $$\partial_{t}S_{S}=D_{S}(\partial_{x}^{2}S_{S}+\partial_{y}^{2}S_{S}+\partial_{z}^{2}S_{S})+r_{S},$$

(4) $$\partial_{t}S_{O}=D_{O}(\partial_{x}^{2}S_{O}+\partial_{y}^{2}S_{O}+\partial_{z}^{2}S_{O})+r_{O},$$

where *D_S_* and *D_O_* are the diffusion coefficients of the nutrient and oxygen, respectively. *r_S_* and *r_O_* are the net reaction rates of the nutrient and oxygen respectively, obtained by summing the rates of all processes involving these respective growth factors. Explicitly, *r_S_* and *r_O_* are expressed by the following equations:

(5) $$r_{S}=-q_{A}\cdot X_{A}-q_{D}\cdot X_{D},$$

(6) $$r_{O}=-(1-Y_{A})q_{A}\cdot X_{A}-(1-Y_{D})q_{D}\cdot X_{D},$$

where *X*_A_ and *X*_D_ are the local biomass densities of active and dormant cells, respectively.

The 3D reaction–diffusion Equations (3–6) are numerically solved under the following three boundary conditions (the coordinate system of the cubic computational space is defined in Supplementary Figure 1B): (a) A Dirichlet boundary condition is imposed along the top boundary, i.e., the concentration remains constant at the interface between boundary layer and bulk liquid. The boundary is defined as the plane *x* = (*N*_bf_ + N_bl_) Δ*x*, where *N*_bf_ and *N*_bl_ are the maximum biofilm thickness and the boundary layer thickness, respectively, expressed in units of grid size (integer), defined as Δ*x* = *L_x_*/*N*. Note that this boundary moves upwards as the biofilm grows over time. This boundary condition is expressed as:

(7) $$\begin{array}{l} S_{S}(x,y,z)=S_{S}^{\text{bulk}}\;and\;S_{O}(x,y,z)=S_{O}^{\text{bulk}},\\ \;for\;x\geq (N_{\text{bf}}+N_{\text{bl}})\Delta x. \end{array}$$

(b) Neumann boundary conditions are imposed at the bottom of the system, where the nutrient and oxygen fluxes are zero:

(8) $$\partial_{x}S_{S}(x=0,y,z)=0\;and\;\partial_{x}S_{O}(x=0,y,z)=0,$$

and (c) periodic boundary conditions are imposed at the lateral boundaries:

(9) $$\begin{array}{l} \;S_{S}(x,y=0,z)\text{ ​​​}=S_{S}(x,y=L_{y},z),\\ \;S_{O}(x,y=0,z)\text{ ​​​}=S_{O}(x,y=L_{y},z),\\ \;S_{S}(x,y,z=0)\;=S_{S}(x,y,z=L_{z}),\\ and\;S_{O}(x,y,z=0)=S_{O}(x,y,z=L_{z}). \end{array}$$

### Implementation of dormant cell formation mechanisms

Four possible mechanisms of dormant cell formation were implemented in the 3D biofilm model. These mechanisms are briefly described below.

- Dormant cell formation by stochastic process: Bacterial cells stochastically enter the dormant state anywhere in the biofilm at constant frequency *R*_*a*→*d*_ (unit = day^−1^) (Chambless et al., 2006).
- Dormant cell formation by a nutrient-dependent process or an oxygen-dependent process: Bacterial cells rarely become dormant cells at high nutrient or oxygen concentration, but readily become dormant at low nutrient or oxygen concentration. The frequency of dormant cell formation by a nutrient-dependent or an oxygen-dependent process is respectively given by:
  (10) $$R_{a\to d}=R_{a\to d}^{\text{max}}exp(-S/K).$$
  where *S* (g/m^3^) is the concentration of nutrient (*S_S_*) or oxygen (*S_O_*), *K* (g/m^3^) is the half saturation constant of nutrient (*K_S_*) or oxygen (*K_O_*), and *R*^max^_*a*→*d*_ (day^−1^) is the maximum frequency of dormancy.
- Dormant cell formation by a time-dependent process: Bacterial cells become dormant when the duration from the last division exceeds some threshold time *T*_*a*→*d*_ (h).

Each mechanism was simulated using the parameter values specified in Table 4. In the simulation, dormant cells are shown in red to visualize their distribution through the biofilm. Dormant cells consume a small amount of nutrient and oxygen for their maintenance without growing. We did not implement the resuscitation of dormant cells in this model because the molecular mechanisms behind the switching back to growth after dormancy are largely unknown.

**Table 4:** **Parameter values of simulated dormant cells**.

**Mechanism of dormancy**	**Parameter**	**Symbol**	**Values used in simulation**	**Unit**
(i) Stochastic process	Frequency of dormancy	*R*_*a*→*d*_	0.01, 0.02, 0.04	day^−1^
(ii.a) Nutrient-dependent process^*^	Half saturation constant of nutrient	*K_S_*	2, 20, 200	gCOD/m^3^
(ii.b) Oxygen-dependent process^*^	Half saturation constant of oxygen	*K_O_*	0.035, 0.35, 3.5	g/m^3^
(iii) Time-dependent process	Threshold time to become dormancy	*T*_*a*→*d*_	10, 12, 14	h

* *In these mechanisms, the maximum frequency of dormancy R^max^_a→d_ was set to 0.12 day^−1^*.

### Numerical simulations

The simulation flow is detailed elsewhere (Picioreanu et al., 2004). Briefly, the initial condition of each simulation (*t* = 0) is ten particles with mass *m*_min_ randomly inoculated along the bottom surface (*x* = 0). In each time step, the spatial distributions of the nutrient and oxygen in the system were obtained by solving Equations (3–9) in steady state. The particles grew, divided, or entered the dormant state as described above. Biofilm formation was simulated for 1 day, or 2 days in cases of slow-growing biofilms. Three simulations with different seeds were conducted for each hypothesis.

### Quantitative analysis of the distribution of dormant cells in biofilm

The composition ratio of the dormant cells along the *x* direction of the biofilm, i.e., the height from the bottom surface, was derived from the simulation results. First, the particles residing at *x* = 0.8*H*, where *H* is the maximum biofilm thickness (in μm), were collected, and the positions (*x*) and states (dormant or active) of all particles below the collected particle were investigated. Precisely, if the collected particle was located at (*x*_P_, *y*_P_, *z*_P_), all particles with centers positioned at (*x*_I_, *y*_I_, *z*_I_) satisfying the following criteria were investigated;

(11) $${\left(y_{I}-y_{P}\right)}^{2}+{\left(z_{I}-z_{P}\right)}^{2}<R_{I}^{2},$$

where *R*_I_ is the radius of the particle. Note that *x*_I_ can be less than 0.8*H*. For each run of the simulation, fifty to two-hundred particles were collected (i.e., these particles resided at *x* = 0.8*H*). Position data of dormant cells below the collected particles were accumulated in three runs of simulations and calculated the abundance of dormant cells for each height level.

## Results and discussion

**Figure 1:**
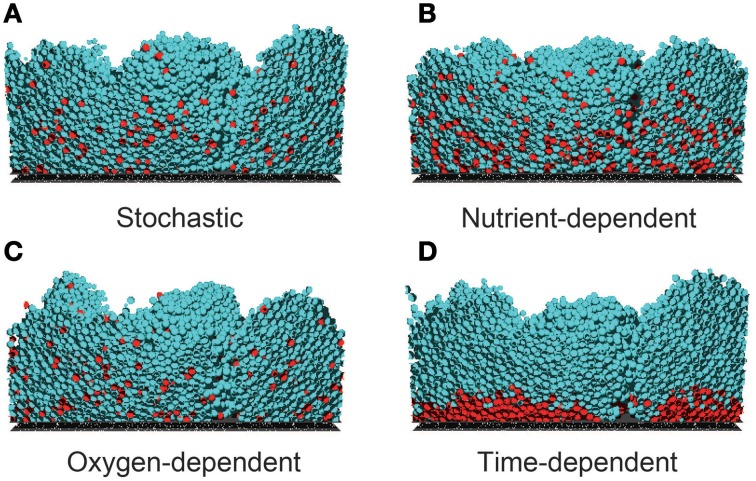
**Cross sections of biofilms 24 h post-inoculation, developed under four dormancy mechanisms**. Dormancy was induced by **(A)** stochastic process (*R*_*a*→*d*_ = 0.04 day^−1^), **(B)** nutrient-dependent process (*K_S_* = 20 gCOD/m^3^), **(C)** oxygen-dependent process (*K_O_* = 3.5 g/m^3^), and **(D)** a time-dependent process (*T*_*a*→*d*_ = 14 h). In the stochastic, nutrient-dependent and oxygen-dependent processes, the dormant cells are widely distributed through the biofilm. In the time-dependent process, dormant cells reside near the bottom of the biofilm. These spatial distributions of dormant cells largely depend on the parameter values related to the dormant cell formation mechanism (see Supplementary Figure 2).

Representative examples of the simulated growing biofilms, assuming each hypothesis of dormant cell formation, are shown in Movies 1–4 (Supplementary Material). Figure 1 shows cross-sections of the biofilms 24 h after inoculation. As shown in this figure, the morphologies of the formed biofilms are very similar under the different hypotheses. Moreover, spatiotemporal dynamics of nutrient and oxygen concentrations within growing biofilms are shown in Movies 5–12 (Supplementary Material). As shown in these Movies, the spatial distributions of nutrient and oxygen are also very similar under the different hypotheses. However, nutrient was completely depleted at the bottom of the biofilm whereas oxygen remained even at the bottom of the biofilm (the value of the oxygen concentration at the bottom of the biofilm was about 2.9 g/m^3^). Therefore, concentration gradients of nutrient and oxygen were different within growing biofilms.

On the other hand, Figure 1 reveals prominent differences in the spatial distributions of dormant cells among the four models. For example, when the cells become dormant by a time-dependent process (Figure 1D), they are restricted to the bottom of the biofilm, whereas they are widely distributed throughout the biofilms in the other three models. This reflects the low growth rate of bacterial cells residing near the bottom of the biofilm, where the nutrient and/or oxygen concentrations are substantially reduced. In fact, when the threshold value *T*_*a*→*d*_ was decreased in the simulations, the region of dormant cells expanded toward the upper region of the biofilm (data not shown). Thus, the size of the dormant region strongly depends on the model parameter values. Moreover, when the half saturation constant of the nutrient *K_S_* was decreased and the maximum frequency of dormant cell formation *R*^max^_*a*→*d*_ was increased in the nutrient-dependent hypothesis, the dormant cell distribution was similar to that of Figure 1D, i.e., dormant cells distributed exclusively near the bottom of the biofilm (Supplementary Figure 2). Thus, the shape of the dormant region is sensitive to the model parameter values.

**Figure 2:**
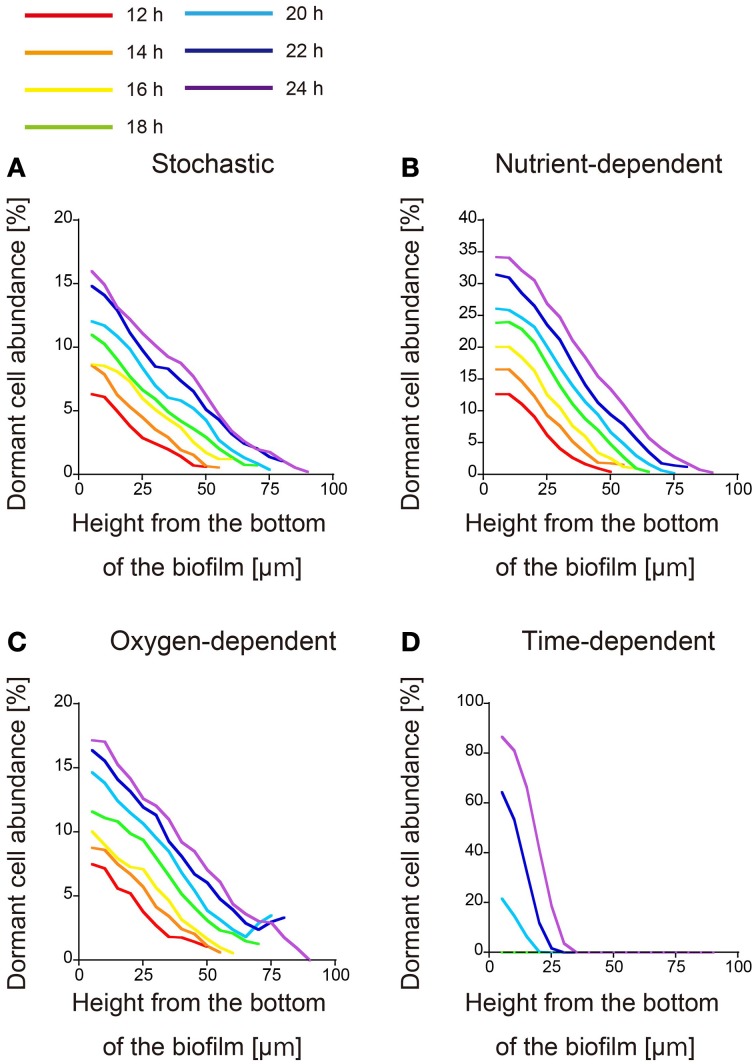
**Time evolution of the spatial distribution of dormant cells**. The abundances of dormant cells are calculated as described in the text and plotted against *x*, the height from the bottom of the biofilm. Dormancy was induced by **(A)** stochastic process, **(B)** nutrient-dependent process, **(C)** oxygen-dependent process, and **(D)** time-dependent process. Each plot was smoothed by moving-average with a 10 μm window (3 points). Distributions obtained at 2-h intervals from 12 to 24 h post-inoculation are plotted in different colors.

Because the parameter values related to dormant cell formation alter the spatial distributions of dormant cells both qualitatively (the shape of the dormant region) and quantitatively (the size of the dormant region), the four hypotheses cannot be discriminated merely from the spatial distributions of dormant cells at a given time. Therefore, we simulated time evolution of the spatial distribution of dormant cells in each of the four proposed models (Figure 2). In this investigation, when dormancy was driven by a time-dependent process (Figure 2D), dormant cells emerged 20 h after inoculation. This process of dormant cell formation was very different from those of the other three models.

All three of the remaining models underwent similar processes of dormant cell formation. Dormant cells were widely distributed throughout the biofilms, and their abundances decreased with increasing height from the bottom of the biofilm (Figures 2A–C). Counter-intuitively, the abundance of dormant cells established a gradient along the *x* direction in the stochastically driven dormancy model, although the probability of dormant cell formation is position-independent in this model (Figure 2A). This gradient probably resulted from the cell velocity gradient along *x*, which was driven by the cell division process. Namely, the velocity in deeper biofilm regions was lower than in higher regions because of the low nutrient and oxygen concentrations. To test this, we conducted the additional simulation under the condition that dormant cells are formed by stochastic process as described below. In the simulation, to cancel the variation in cell velocities along the *x* direction, we reset *K_S_* and *K_O_* to be very small values. The results showed that the abundance of dormant cells did not establish a gradient along the *x* direction (Supplementary Figure 3). Therefore, the variation in the cell velocities along the *x* direction is responsible for the gradient of the abundance of dormant cells in the stochastically driven dormancy model.

**Figure 3:**
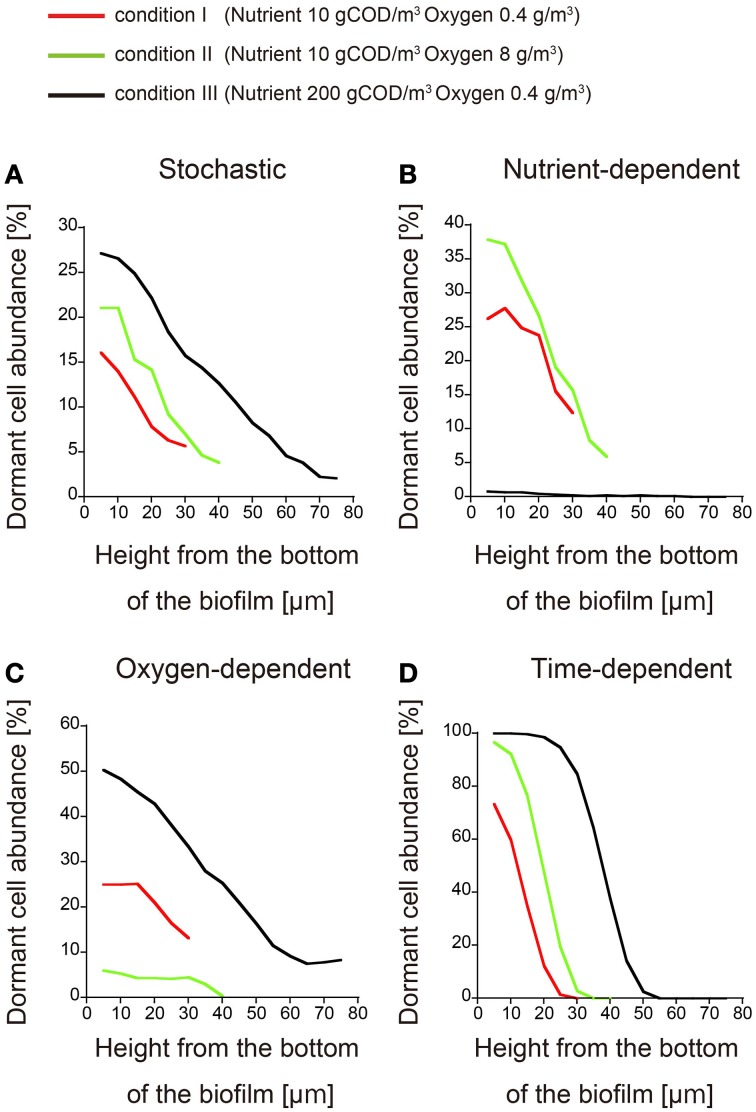
**Spatial distributions of dormant cells obtained for different nutrient and oxygen concentrations in the bulk**. The abundances of dormant cells are plotted against the biofilm height under conditions I (red), II (green), and III (black) (the conditions are defined in the text). Dormancy was induced by **(A)** stochastic process, **(B)** nutrient-dependent process, **(C)** oxygen-dependent process, and **(D)** time-dependent process. Each plot was smoothed by moving-average with a 10 μm window (3 points).

To confirm the difference between these three models, we can investigate how the dormant cell distribution responds to an increase or decrease in the nutrient and oxygen concentrations in the bulk liquid. Such an investigation should be simple and straightforward. Thus, biofilm formation was simulated under three conditions of nutrient and oxygen concentrations in the bulk liquid (expressed in units of gCOD/m^3^ and g/m^3^, respectively): (condition I) 10 gCOD/m^3^ and 0.4 g/m^3^, (condition II) 10 gCOD/m^3^ and 8 g/m^3^, and (condition III) 200 gCOD/m^3^ and 0.4 g/m^3^. When dormant cell formation was induced by stochastic or time-dependent processes, the dormant cell distribution was not qualitatively affected by altering the bulk concentrations of nutrient and oxygen, i.e., there was a height gradient in the abundance of dormant cells under all three conditions (Figures 3A,D). Conversely, if dormancy was induced by a nutrient-dependent process, dormant cells rarely emerged in the biofilm at very high nutrient concentration (200 gCOD/m^3^; condition III) (Figure 3B). Similarly, in the oxygen-dependent model, dormant cells rarely emerged when the oxygen concentration was high (8 g/m^3^; condition II) (Figure 3C). Therefore, these three models can be discriminated by investigating their qualitative responses to altered bulk concentrations of nutrient and oxygen.

As shown in Figures 1, 2, the dormant cells resided in the deeper region of biofilms in the present four simulation models. There were several previous studies investigating the actual spatial distributions of dormant cells within biofilms. In one study, colony biofilms of *P. aeruginosa* PAO1 containing a plasmid with an isopropylthio-β-D-galactoside (IPTG) inducible GFP gene were cultured on polycarbonate membranes placed on LB agar plates. After 48 h cultivation, the membranes were transferred to an LB plate containing IPTG for an additional 4 h. The IPTG induced GFP expression in the active cells. The GFP-expressing cells were observed only in the upper region of the colony biofilm, although the nutrient was supplied through the membrane at the bottom of the colony biofilm (Kim et al., 2009). Williamson et al. (2012) also performed similar experiments using a continuous-drip flow biofilm system which nutrient enter from above and obtained similar results. This result implies that dormant cells reside in the deeper regions of biofilms, which is consistent with our simulation results. The spatial distribution of dormant cells in biofilms obtained in the above reports can reject none of the four hypotheses on the mechanisms of dormant cell formation implemented into the computational models developed in the present study. As stated above, time evolution of the distribution and/or the responses to the increase/decrease of the substrate/oxygen may provide novel insights about the mechanism of dormancy.

## Conclusions

To predict the formation of dormant cells in growing biofilms, we proposed four hypotheses of dormant cell formation and implemented them in a 3D biofilm model based on the IbM algorithm. In numerical simulations of the model, we found that (i) in the stochastic hypothesis of dormant cell formation, an unexpected gradient appeared in the abundance of dormant cells along the depth direction; thus (ii) investigating the spatial distributions of dormant cells at a specific time cannot discriminate among the four suggested dormancy mechanisms; however, (iii) all four hypotheses were discriminated in spatiotemporal studies of the dormant cell distributions while varying the bulk concentrations of nutrient and/or oxygen. The proposed simulation methodology could guide experiments for efficiently elucidating the mechanisms of dormant cell formation in growing biofilms.

The following steps are the method to determine what kinds of experimental studies are effective for discriminating dormant cell formation mechanisms in biofilms. First, we will establish the biofilm into which nutrients enter from above in liquid medium, for example, a continuous-drip flow biofilm system, and should investigate the spatial distribution of the dormant cell within growing biofilm. At that time, we should use the detecting system of dormant cell, such as degradable GFP (half-life < 1 h) under the control of the ribosomal *rrn*BP1 promoter, which normally controls expression of the *rrnB* gene which codes for 16S rRNA (Shah et al., 2006; Maisonneuve et al., 2013), or TIMER^bac^ fluorescence system in which both rapidly maturing green and slowly maturing orange TIMER molecules can accumulate, whereas in dividing cells, rapidly maturing green molecules dominate over orange molecules that are diluted by cell division (Claudi et al., 2014). Second, we will observe time evolution of the spatial distribution of dormant cells in growing biofilm by measurement with the use of microscopy. In this observation, if dormant cell arise near the bottom of the biofilm good long time after inoculation, a time-dependent process will be a plausible mechanism of dormancy (Figure 2D). Third, if dormant cells are widely distributed throughout the biofilm, we should investigate the responses to the increase/decrease of the nutrient/oxygen. If the mechanism of dormant cell formation was caused by stochastic process, the distribution of dormant cells in biofilms was not affected qualitatively by the change in the bulk concentrations of the nutrient and oxygen (Figure 3A). On the other hand, if the mechanism was caused by the nutrient-dependent process, dormant cells rarely emerged in the biofilm when the concentration of the nutrient was very high (condition III) (Figure 3B). Similarly, if the mechanism was caused by oxygen-dependent process, dormant cells rarely emerged when the concentration of oxygen is high (condition II) (Figure 3C). As stated above, we could find a clue of the dynamics of dormant cell formation within growing biofilm by comparing the simulation results provided in present study with experimental results.

In summary, the simulation results of this study suggest that, by experimentally investigating the spatiotemporal dormant cell distributions while varying the nutrient and oxygen concentrations in the bulk, we could gain new insights into how dormant cell populations establish in biofilms.

### Conflict of interest statement

The authors declare that the research was conducted in the absence of any commercial or financial relationships that could be construed as a potential conflict of interest.
