# Supplementary material for: Mathematical modeling of dormant cell formation in growing biofilm
Source: Front Microbiol. 2015 May 28;6:534. doi: 10.3389/fmicb.2015.00534 (PMC4446547; doi:10.3389/fmicb.2015.00534)
Supplement: Supplementary file 13 [file DataSheet1.DOCX]

***Supplementary Material***

**Mathematical modeling of dormant cell formation**

**in growing biofilm**

**Kotaro Chihara^1,¶^, Shinya Matsumoto^2, ¶^, Yuki Kagawa^3, ¶^, Satoshi Tsuneda^1,3,*^**

^1^ Department of Life Science and Medical Bioscience, Waseda University, 2-2 Wakamatsu-cho, Shinjuku, Tokyo 162-8480, Japan

^2^ Center for Biofilm Engineering, Montana State University, Bozeman, Montana 59717-3980, USA

^3^ Institute for Nanoscience and Nanotechnology, Waseda University, 2-2 Wakamatsu-cho, Shinjuku, Tokyo 162-8480, Japan

^¶^ These three authors contributed equally to this work.

**^*^ Correspondence:** Satoshi Tsuneda, Department of Life Science and Medical Bioscience, Waseda University, 2-2 Wakamatsu-cho, Shinjuku, Tokyo 162-8480, Japan

Tel/Fax: +81 3 5369 7325.

E-mail: stsuneda@waseda.jp

1. **Legends of Movies**

**Movie 1.** Representative simulation result of growing biofilm obtained from the model adopting the stochastic mechanism of cell dormancy.

**Movie 2.** Representative simulation result of growing biofilm obtained from the model adopting the nutrient-dependent mechanism of cell dormancy.

**Movie 3.** Representative simulation result of growing biofilm obtained from the model adopting the oxygen-dependent mechanism of cell dormancy.

**Movie 4.** Representative simulation result of growing biofilm obtained from the model adopting the time-dependent mechanism of cell dormancy.

**Movie 5.** Concentration gradient of nutrient every 4 h after inoculation obtained from the model adopting the stochastic mechanism of cell dormancy.

**Movie 6.** Concentration gradient of oxygen every 4 h after inoculation obtained from the model adopting the stochastic mechanism of cell dormancy.

**Movie 7.** Concentration gradient of nutrient every 4 h after inoculation obtained from the model adopting the nutrient-dependent mechanism of cell dormancy.

**Movie 8.** Concentration gradient of oxygen every 4 h after inoculation obtained from the model adopting the nutrient-dependent mechanism of cell dormancy.

**Movie 9.** Concentration gradient of nutrient every 4 h after inoculation obtained from the model adopting the oxygen-dependent mechanism of cell dormancy.

**Movie 10.** Concentration gradient of oxygen every 4 h after inoculation obtained from the model adopting the oxygen-dependent mechanism of cell dormancy.

**Movie 11.** Concentration gradient of nutrient every 4 h after inoculation obtained from the model adopting the time-dependent mechanism of cell dormancy.

**Movie 12.** Concentration gradient of oxygen every 4 h after inoculation obtained from the model adopting the time-dependent mechanism of cell dormancy.

1. **Supplementary Figures**

**
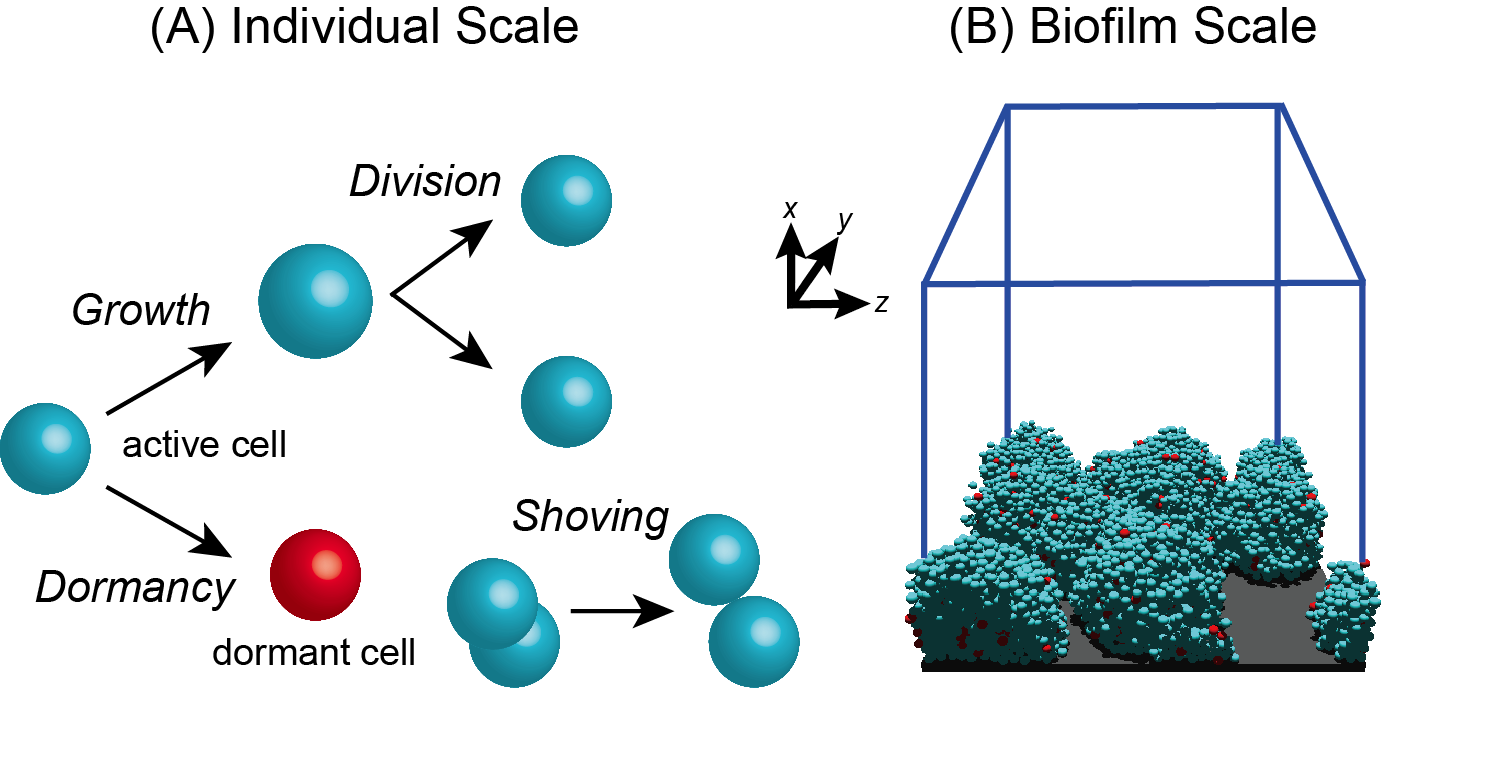
**

**Supplementary Figure 1. Model system.** (A) Each bacterial cell (blue spheres), grows, divides into two cells, and shoves other cells to minimize overlaps. A portion of the active cells shows the dormant phenotype (red spheres). (B) Simulated biofilm formation originated from ten cells randomly inoculated onto the bottom of the cubic computational space. Boundary conditions of the computational space are described in the main text.

**
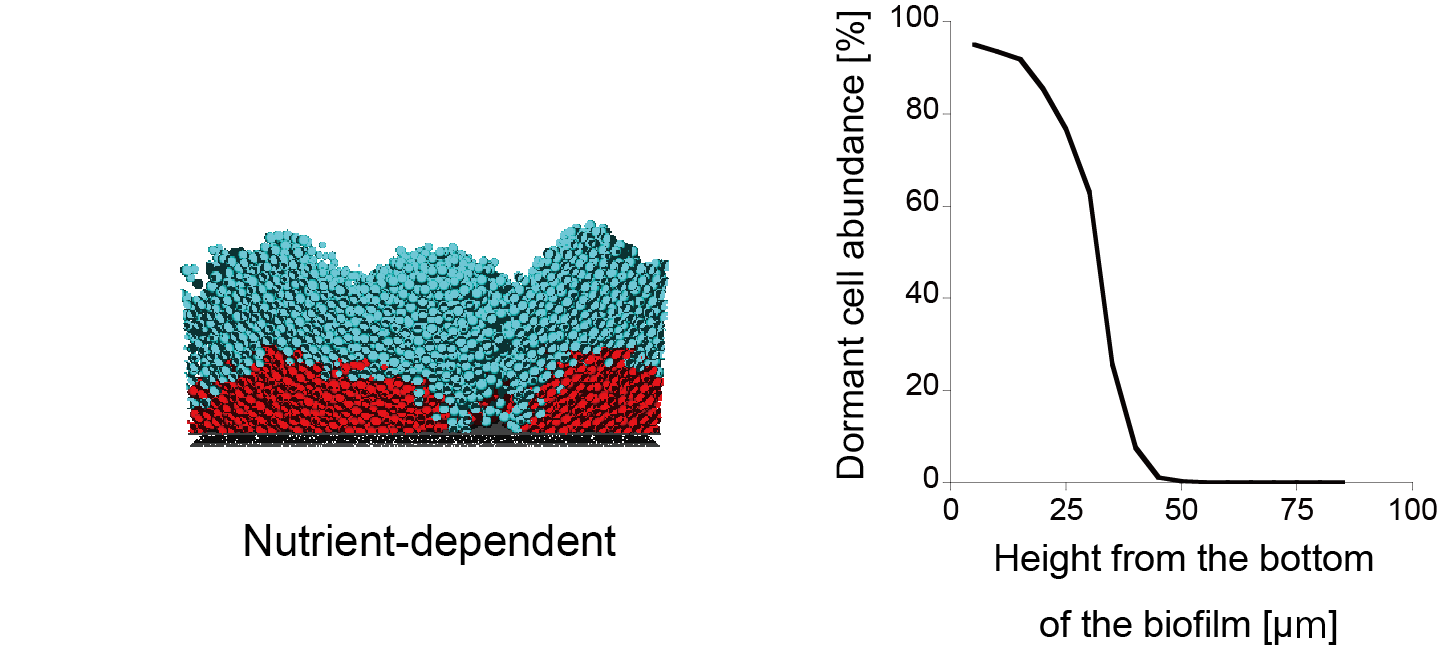
**

**Supplementary Figure 2. The distribution of dormant cells in the biofilm strongly depends on the parameter values related to dormant cell formation.** In this example, the dormant cell distribution can be qualitatively altered by changing the parameter values. The dormant cells were widely distributed in the nutrient-dependent mechanism of cell dormancy (see main text, Fig. 1B); however, when the parameter *R*_a→d_^max^ was increased from 0.12 to 24 day^-1^, while *K_S_* was reduced from 20 to 0.002 gCOD/m^3^, the above distribution was obtained. Shown are a cross section (left) and the spatial distribution of the dormant cells (right) at *t* = 24 h.


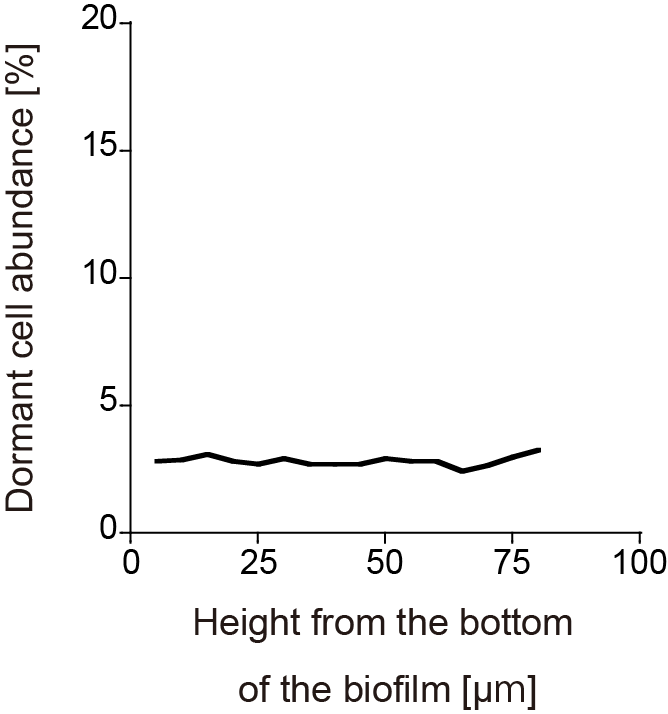


**Supplementary Figure 3. A gradient of dormant cell abundance along the biofilm height was ceased when the growth rates were position independent in the stochastically driven dormancy model.** In this example, we set *K_S_* and *K_O_* to be 1.0 × 10^-20^ and 1.0 × 10^-20^, respectively, to remove the position dependency of the cell velocity in the stochastically driven dormancy model with *R*_a→d_ = 0.04 day^-1^.　Distribution obtained at 24 h post inoculation is plotted.
